# Deterministic Input, Noisy Mixed Modeling for Identifying Coexisting Condensation Rules in Cognitive Diagnostic Assessments

**DOI:** 10.3390/jintelligence11030055

**Published:** 2023-03-16

**Authors:** Peida Zhan

**Affiliations:** 1 School of Psychology, Zhejiang Normal University, Jinhua 321004, China; zhan@zjnu.edu.cn; 2 Intelligent Laboratory of Child and Adolescent Mental Health and Crisis Intervention of Zhejiang Province, Jinhua 321004, China; 3 Key Laboratory of Intelligent Education Technology and Application of Zhejiang Province, Jinhua 321004, China

**Keywords:** cognitive diagnosis, condensation rule, cognitive diagnosis models, DINMix model

## Abstract

In cognitive diagnosis models, the condensation rule describes the logical relationship between the required attributes and the item response, reflecting an explicit assumption about respondents’ cognitive processes to solve problems. Multiple condensation rules may apply to an item simultaneously, indicating that respondents should use multiple cognitive processes with different weights to identify the correct response. Coexisting condensation rules reflect the complexity of cognitive processes utilized in problem solving and the fact that respondents’ cognitive processes in determining item responses may be inconsistent with the expert-designed condensation rule. This study evaluated the proposed deterministic input with a noisy mixed (DINMix) model to identify coexisting condensation rules and provide feedback for item revision to increase the validity of the measurement of cognitive processes. Two simulation studies were conducted to evaluate the psychometric properties of the proposed model. The simulation results indicate that the DINMix model can adaptively and accurately identify coexisting condensation rules, existing either simultaneously in an item or separately in multiple items. An empirical example was also analyzed to illustrate the applicability and advantages of the proposed model.

## 1. Introduction

Existing cognitive diagnosis models (CDMs) and diagnostic classification models (e.g., Rupp et al. 2010; von Davier and Lee 2019) can be classified into three categories based on the condensation rule of how latent attributes (e.g., skills, knowledge, and cognitive processes) influence respondents’ observed item responses: conjunctive, disjunctive, and compensatory (Maris 1995, 1999). Models with the conjunctive condensation rule, such as the deterministic input, noisy ‘and’ gate (DINA) model (Junker and Sijtsma 2001), assume that respondents must master all the required attributes of an item to provide the correct response. In contrast, models with the disjunctive condensation rule, such as the deterministic input, noisy ‘or’ gate (DINO) model (Templin and Henson 2006), assume that respondents can provide the correct response to an item if they have mastered any of the required attributes. Furthermore, models with the compensatory condensation rule, such as the additive CDM (ACDM) model (de la Torre 2011), assume that for the correct response probability, the mastery of a particular attribute may compensate for the non-mastery of another attribute.

Applying a CDM should be based on a suitable condensation rule, usually designed by experts during the test/item development phase. Condensation rules designed by experts define the theoretical relationship between the measured attributes and the observed item responses, reflecting an explicit assumption about the cognitive processes engaged by respondents during problem solving. However, in practice, respondents’ cognitive processes in their item responses may be inconsistent with the expert-designed condensation rule. This phenomenon indicates that the validity of the measurement of cognitive processes is low and has been ignored by most previous studies.

In addition to reduced CDMs with a specific condensation rule, general CDMs involving saturated interaction terms have also been developed, such as the generalized DINA model (GDINA; de la Torre 2011), log-linear CDM (LCDM; Henson et al. 2009), and general diagnostic model (GDM; von Davier 2008a). General CDMs account for the complexity of cognitive processes from the statistical analysis perspective.^1^ They are, therefore, not limited to a specific condensation rule, which increases their theoretical application scope. However, this ‘rough’ approach causes general CDMs to obscure the theoretical relationship between the required attributes and the item response, as it sidesteps the validity of the measurement of cognitive processes. When general CDMs are used, it is not easy to gain a clear understanding of how the required attributes affect correct response probability, which reduces the interpretability of diagnostic results, especially in relation to cognitive processes.

Compared to general CDMs, reduced CDMs may be more appropriate in practice for four reasons. First, reduced CDMs usually require smaller sample sizes for accurate and robust parameter estimation (cf. Jiang and Ma 2018). Second, the simpler model is preferred if its performance is not significantly worse than the more complex model, according to the parsimony principle (e.g., Beck 1943). Third, item parameters in reduced CDMs typically have more straightforward interpretations, which makes working with them attractive to practitioners (Liu et al. 2009). Fourth, reduced models reflect the cognitive processes engaged in solving problems more clearly, increasing diagnostic results’ interpretability.

To balance the general and reduced models, de la Torre and Lee (2013) and Ma et al. (2016) proposed the Wald test approach for selecting a suitable reduced model from the GDINA model for each item. This approach assumes that each item belongs to only one appropriate condensation rule, for example, the conjunctive or the disjunctive. However, after using this method in practice, one might realize that its results may indicate that the required attributes in an item may satisfy multiple condensation rules simultaneously (i.e., coexisting condensation rules in an item). Specifically, this can be observed in two phenomena. No reduced model applies to some items except the general model, which indicates that a specific condensation rule cannot simply describe the relationship between the required attributes and the item response. For some other items, there is more than one reduced model with insignificant differences from the general model. Such results indicate that multiple condensation rules may be simultaneously applied to the same item and only differ in degree. These two phenomena can be found when using the Wald test to the data contained in some previous studies (e.g., Ma et al. 2016; Jang 2009; Ravand et al. 2013), and they indicate that the correct response to an item may have uncertainty regarding the requirement of cognitive processes. From the statistical analysis perspective, if such uncertainty can be considered in modeling, it may increase the degree of model-data fitting, thus improving diagnostic accuracy. From the measurement perspective, if we can identify such uncertainty and feed it back into item revision to ensure that the cognitive process used by respondents matches the expert-designed cognitive process, it would help improve the validity of the measurement of cognitive processes.

Unlike general CDMs, which ‘roughly’ introduce saturated interaction terms to consider the potential for coexisting condensation rules, the current study aims to identify coexisting condensation rules to provide feedback for item revision. Inspired by but different from mixture/hybrid item response models (e.g., Man and Harring 2022; Mislevy and Verhelst 1990; von Davier 2008b; Yamamoto 1989) and multi-strategy CDMs (e.g., de la Torre and Douglas 2008; Ma and Guo 2019), a new model called the deterministic inputs, noisy mixed (DINMix) model is proposed. The proposed model allows each item to simultaneously contain multiple condensation rules, thus realizing the identification of different ones. Furthermore, the item response function of the proposed model is more concise than that of the general CDMs, which also increases the interpretability of item parameters to some extent, making it more attractive to practitioners.

The remaining sections of this paper are constructed as follows. First, deterministic input, noisy models with three different condensation rules, and mixture item response models are briefly reviewed. Next, the proposed DINMix model is presented, followed by two simulation studies that evaluate the psychometric properties of the proposed model. An empirical example is then analyzed to illustrate the applicability and advantages of the proposed model. Finally, the paper concludes with a discussion of the limitations of the proposed model and suggestions for further research.

## 2. Background

### 2.1. Deterministic Input, Noisy Models with Typical Condensation Rules

In psychometric models, a commonly used item response function of the relationship between observed and latent responses can be expressed as follows:

(1) $$P\left(y_{ni}=1|g_{i},\;s_{i},\;\omega_{ni}\right)=g_{i}+\left(1-s_{i}-g_{i}\right)\omega_{ni}$$

where *y_ni_* is the observed (dichotomous) response of person *n* (*n* = 1, …, *N*) to item *i* (*i* = 1, .., *I*); $\omega_{ni}$ is the latent response of person *n* to item *i*; *g_i_* and *s_i_* are the guessing and slip parameters of item *i*, respectively. Typically, a monotonicity restriction, *g_i_* < 1 − *s_i_*, can be imposed (Junker and Sijtsma 2001; Culpepper 2015). With various choices of $\omega_{ni}$, Equation (1) can describe many psychometric models, such as the four-parameter logistic unidimensional or multidimensional item response models (Reckase 2009) and the multicomponent latent trait model (Embretson 1984).

In CDMs, the latent response, $\omega_{ni}$, is the concrete expression of the condensation rule. When assuming that attributes follow the conjunctive condensation rule, the latent response has

(2) $$\omega_{ni}=\prod_{k=1}^{k}\alpha_{nk}^{q_{ik}}$$

where α*_nk_* is the mastery status of person *n* of attribute *k* (*k* = 1, …, *K*), α*_nk_* = 1 means mastery, and α*_nk_* = 0, otherwise. The Q-matrix (Tatsuoka 1983) is an *I*-by-*K* matrix with element *q_ik_* indicating whether attribute *k* is required to answer item *i* correctly; *q_ik_* = 1 if the attribute is required, and *q_ik_* = 0 otherwise. With the conjunctive condensation rule, Equation (1) becomes the DINA model.

In contrast, when assuming that the attributes follow the disjunctive condensation rule, the latent response has

(3) $$\omega_{ni}=1-\prod_{k=1}^{k}{\left(1-\alpha_{nk}\right)}^{q_{ik}}$$

and Equation (1) becomes the DINO model.

Conjunction and disjunction are two extreme condensation rules, and both divide individuals into two groups: full (non)mastery and partial mastery. These rules do not further differentiate individuals in the partial mastery group. To mitigate this issue, the ratio condensation rule can be adopted, where

(4) $$\omega_{ni}=\frac{\sum_{k=1}^{K}\alpha_{nk}q_{ik}}{\sum_{k=1}^{K}q_{ik}}$$

With the ratio condensation rule, Equation (1) becomes the deterministic input, noisy ratio (DINR) model. The ratio condensation rule is a special compensatory condensation rule that assumes increases in the number of mastered attributes are linearly related to increases in correct response probability. In such cases, the DINR model can be considered as a particular case of the ACDM with the equal main effect of each attribute (i.e., $\left(1-s_{i}-g_{i}\right)/\overset{K}{\underset{k=1}{\sum }}q_{ik}$). Thus, the DINR model assumes that all required attributes are equally weighted. The DINR model is considered instead of the ACDM to avoid introducing additional item parameters, which keeps the model as simple as possible.

### 2.2. Mixture Item Response Models

Mixture/hybrid item response models are well studied and primarily used for detecting and explaining the differential behavior of individuals of multiple latent groups in the population (i.e., population heterogeneity). Typically, in mixture item response models, multiple latent groups of individuals are assumed to be present in the sampled population. These models have been widely applied to diverse research issues, such as identifying problem-solving strategies (Mislevy and Verhelst 1990; Wang and Xu 2015) and detecting differential item functioning (Cohen and Bolt 2005). Furthermore, for diagnostic classification purposes, von Davier (2008b) proposed the GDM mixture, which extends the GDM to tests with multiple observed and latent groups. Choi (2010) proposed a diagnostic classification mixture Rasch model, incorporating the mixture Rasch model (Rost 1990) and the LCDM. Yamaguchi and Okada (2020) presented a hybrid CDM, defined as a weighted mixture of the DINA and DINO models. This model assumes two latent groups in the population; one group of respondents’ responses are suitable for analysis using the DINA model, and the other group’s responses are suitable for analysis using the DINO model. However, the distinguishing feature of the proposed model in this study is that mixture modeling accommodates the simultaneous existence of multiple condensation rules rather than multiple latent groups of individuals.

## 3. Deterministic Input, Noisy Mixed Model

### 3.1. Model Formulation

As stated previously, this study aims to present a new CDM that can incorporate and identify coexisting condensation rules. In the current study, we interpret the coexisting condensation rules within an item as the required attributes that affect individuals’ item responses according to multiple condensation rules with different proportions. Thus, when an item contains coexisting condensation rules, respondents should use multiple cognitive processes with different weights to respond correctly to this item.

Inspired by but different from the mixture item response models, the item response function of the DINMix model can be expressed as

(5) $$P\left(y_{ni}=1|g_{i},\;s_{i},\;\Psi_{ni}\right)=g_{i}+\left(1-s_{i}-g_{i}\right)\Psi_{ni}$$

where $\Psi_{ni}$ is the mixed latent response of person *n* to item *i*, which can be further defined as

(6) $$\Psi_{ni}=\sum_{m=1}^{M_{i}}\tau_{im}\omega_{nim}$$

where $\omega_{nim}$ is the latent response of person *n* to item *i* in condensation rule *m* (*m* = 1, …, *M_i_*); $\tau_{im}$ is the item-level mixing proportion parameter of condensation rule *m* in item *i*, which satisfies $\tau_{im}\in \left[0\;,1\right]$ and $\overset{M_{i}}{\underset{m=1}{\sum }}\tau_{im}=1$ for each item. The item-level mixing proportion parameters can be interpreted as the proportions/weights of different cognitive processes required to solve the problem correctly. *M_i_* is the number of preselected condensation rules for item *i*. For simplicity, but without loss of generality, it can be assumed that *M_i_* = *M* for all items throughout this study.

In this study, three typical condensation rules are considered for each item: conjunctive (see Equation (2)), disjunctive (see Equation (3)), and ratio (see Equation (4)) condensation rules. Thus, Equation (6) can be further expressed as

(7) $$\Psi_{ni}=\tau_{i1}\prod_{k=1}^{k}\alpha_{nk}^{q_{ik}}+\tau_{i2}\left(1-\prod_{k=1}^{k}{\left(1-\alpha_{nk}\right)}^{q_{ik}}\right)+\tau_{i3}\frac{\sum_{k=1}^{K}\alpha_{nk}q_{ik}}{\sum_{k=1}^{K}q_{ik}}$$

Finally, the DINMix model can be expressed as

(8) $$\begin{array}{c} P\left(y_{ni}=1|g_{i},\;s_{i},\;\tau_{i},\;\alpha_{nk},\;q_{ik}\right)\\ \;=g_{i}+\left(1-s_{i}-g_{i}\right)\left(\tau_{i1}\prod_{k=1}^{k}\alpha_{nk}^{q_{ik}}+\tau_{i2}\left(1-\prod_{k=1}^{k}{\left(1-\alpha_{nk}\right)}^{q_{ik}}\right)+\tau_{i3}\frac{\sum_{k=1}^{K}\alpha_{nk}q_{ik}}{\sum_{k=1}^{K}q_{ik}}\right) \end{array}$$

where all parameters have been defined above.

Equation (8) is an example of the DINMix model with three typical condensation rules. In practice, by considering uncertainty during the item development phase, experts can assist in determining which condensation rules may be included in the DINMix model for each item in a particular test. For example, when any two of the three condensation rules are preselected by experts, Equation (8) can be reduced to another mixed model with only two condensation rules. Furthermore, the DINA, DINO, and DINR models can all be viewed as special cases of the DINMix model by preselecting a specific condensation rule for all items. Suppose it is difficult for experts to preselect all the possible condensation rules for a particular item. In that case, Equation (8) can be used to identify potential condensation rules from the data-driven perspective.

Theoretically, the number of item parameters in the DINMix model is 2*I +* (*M* − 1)*I*, where *M* = 3 in this study. However, when an item requires only one attribute, there are no differences among all the condensation rules. Thus, an additional constraint can be added to the DINMix model to reduce the number of item parameters, which means that for unidimensional items, the test-level mixing proportion parameters are set as **τ***_i_* = (τ*_i_*_1_, τ*_i_*_2_, τ*_i_*_3_)’ ≡ (1, 0, 0), indicating that the DINA model is forcibly assigned to unidimensional items.^2^

The concept of coexisting condensation rules is more difficult to interpret than a single one. Although coexisting condensation rules describe the complex relationship between the required attributes and the item response, they reduce the interpretability of the model parameters compared to reduced models. The interpretable and meaningful insights gained from the model are essential in educational and psychological applications to meet the need for accountability (Zhan 2020). Thus, using the proposed model to identify items containing coexisting condensation rules is of considerable practical significance. It may improve the degree of model-data fitting, thus improving diagnostic accuracy, as well as help guide item revision to make revised items contain a single expert-defined condensation rule, thereby improving the validity of the measurement and increasing the interpretability of the model parameters.

### 3.2. Bayesian Parameter Estimation

The Bayesian Markov chain Monte Carlo (MCMC) method is used to estimate model parameters. This study used JAGS (version 4.3.0) software to automate the estimation process. The corresponding code for the DINMix model and the other models used in this study are available at https://osf.io/s2yjv/ (accessed on 15 March 2023). More details about using JAGS for Bayesian CDM estimation are provided in a tutorial by Zhan et al. (2019a).

### 3.3. Relationship with Existing CDMs

With three typical and representative condensation rules, the DINMix model is sufficient to cover various relationships between the required attributes and the item response in most cases. Thus, the DINMix model is more general than the aforementioned reduced models. As previously stated, the DINA, DINO, and DINR models can all be viewed as special cases of the DINMix model by preselecting a specific condensation rule.

The differences among the four deterministic inputs, noisy models with the different condensation rules described above are listed in Table 1, along with the item correct response probabilities of eight individuals with different attribute profiles for an item (**q***_i_* = (1, 1, 1), *g_i_* = *s_i_* = .1, τ*_i_*_1_ = .8, τ*_i_*_2_ = .1) based on them. First, compared to the DINA and DINO models, the DINR and DINMix models can better reflect the differences in various attribute profiles. Second, the values of item-level mixing proportions indicate that this item contains three condensation rules simultaneously, and the proportion of the conjunctive condensation rule is the highest. The DINMix model not only differentiates individuals in the partial mastery group but also reflects the feature of the conjunctive condensation rule (i.e., individuals in the partial mastery group have low correct response probabilities).

Additionally, although the DINMix model was initially developed for adaptively identifying coexisting condensation rules, it can still be viewed as a constraint model of the GDINA model after some parameter transformations (see Section S1 in the Supplementary). For each within-item multidimensional item, the number of item parameters in the GDINA model increases as the number of required attributes increases, while the number of item parameters in the DINMix model is always four (i.e., *s_i_*, *g_i_*, τ*_i_*_1_, and τ*_i_*_2_). Therefore, similar to the DINA model, all items in the DINMix model have the same number of item parameters that are easier to interpret than those (e.g., main effects, two-way interactions, three-way interactions) in the GDINA model. More importantly, the DINMix and GDINA models were developed for different purposes. The former was designed for identifying coexisting condensation rules, and the latter was created for model generalization.

### 3.4. Parameter Identifiability

Parameter identifiability is an essential issue in CDMs. It is necessary for consistently estimating model parameters and valid statistical inferences (e.g., Gu and Xu 2019, 2020). As mentioned above, the DINMix model can be viewed as a constraint model of the GDINA model. According to Gu and Xu’s (2019) classification of CDMs, the DINMix model is a multi-parameter Q-restricted latent class model. Thus, the conditions for generic identifiability (i.e., conditions C5 and C6 in Theorem 7), which were also given by Gu and Xu (2019), also apply to the DINMix model.

## 4. Simulation Studies

Two simulation studies were conducted. The purpose of simulation Study 1 was to determine whether parameters of the DINMix model could be recovered accurately, especially whether the proposed model can correctly identify items that contain coexisting condensation rules, in which the data were simulated from the DINMix model and analyzed. Simulation Study 2 was conducted to compare the performance of the proposed model and some other CDMs in six simulated test situations to illustrate the relative advantages and disadvantages of the proposed model.

### 4.1. Study 1

#### 4.1.1. Design and Data Generation

In simulation Study 1, five factors were manipulated, including (a) sample size (*N*) at two levels, 500 and 1000, (b) test length (*I*) at two levels, 15 and 30, and (c) item quality (*IQ*) at two levels, higher and lower. Referencing Zhan et al. (2019b), item parameters were generated from a bivariate normal distribution with a negative correlation coefficient using

(9) $$\left(\begin{array}{c} logit\left(g_{i}\right)\\ logit\left(s_{i}\right) \end{array}\right)\sim MVN\left(\left(\begin{array}{c} -2.197\\ -2.197 \end{array}\right),\left(\begin{array}{cc} 1 & \\ -0.6 & 1 \end{array}\right)\right)$$

for higher-quality items and

(10) $$\left(\begin{array}{c} logit\left(g_{i}\right)\\ logit\left(s_{i}\right) \end{array}\right)\sim MVN\left(\left(\begin{array}{c} -1.386\\ -1.386 \end{array}\right),\left(\begin{array}{cc} 1 & \\ -0.6 & 1 \end{array}\right)\right)$$

for lower-quality items. These settings led to the guessing and slip probabilities for all items following a positively skewed distribution (mean ≈ 0.1, minimum ≈ 0.01, and maximum ≈ 0.6 for higher quality, and mean ≈ 0.2, minimum ≈ 0.05, and maximum ≈ 0.7 for lower quality), assuming that guessing and slip parameters follow a negative correlation is more realistic (Zhan et al. 2019b). Additionally, (d) the type of item-level mixing proportion (*TM*) at two levels of uniform and skew mixing was manipulated. Since the first *I*/3 items in the Q-matrix are unidimensional items, we set **τ**_1~*I*/3_ = (1, 0, 0). Then, we set τ(*I*/3 + 1)~*I* = (1/3, 1/3, 1/3) for uniform mixing. In contrast, for skew mixing, we set **τ**_(*I*/3+1)~8*I*/15_ = (0.6, 0.2, 0.2), **τ**_(8*I*/15+1)~11*I*/15_ = (0.2, 0.6, 0.2), and **τ**_(11*I*/15+1)~*I*_ = (0.2, 0.2, 0.6). Furthermore, (e) the latent structural model of attributes (*LSM*) at two levels of an unstructured and a multivariate normal distribution was manipulated. When an unstructured *LSM* was used, the true attribute profile of each person was randomly chosen from all possible patterns with equal probability; in these cases, the tetrachoric correlations among attributes were approximately zero. In contrast, when a multivariate normal distribution was used, a latent variable matrix with continuous elements was first generated from a five-dimensional multivariate normal distribution (e.g., Chiu et al. 2009):

(11) $$\Theta =\left(\begin{array}{c} \begin{array}{c} \theta_{1}\\ \theta_{2} \end{array}\\ \begin{array}{c} \theta_{3}\\ \theta_{4}\\ \theta_{5} \end{array} \end{array}\right)\sim MVN\left(\left(\begin{array}{c} \begin{array}{c} 0\\ 0 \end{array}\\ \begin{array}{c} 0\\ 0\\ 0 \end{array} \end{array}\right),\left(\begin{array}{c} \begin{array}{c} 1\\ 0.6 \end{array}\\ \begin{array}{c} 0.6\\ 0.6\\ 0.6 \end{array} \end{array}\;\begin{array}{c} \begin{array}{c} 1 \end{array}\\ \begin{array}{c} 0.6\\ 0.6\\ 0.6 \end{array} \end{array}\;\begin{array}{c} \begin{array}{c} 1\\ 0.6\\ 0.6 \end{array}\; \end{array}\;\begin{array}{c} \begin{array}{c} 1\\ 0.6 \end{array}\; \end{array}\;\begin{array}{c} \begin{array}{c} 1 \end{array} \end{array}\right)\right)$$

where $\theta_{k}=\left(\theta_{1k},\;\ldots ,\theta_{Nk}\right)’$; then, the true attribute was determined by

(12) $$\alpha_{nk}=\{\begin{array}{c} \begin{array}{cc} 0 & \theta_{nk}<0 \end{array}\\ \begin{array}{cc} 1 & \theta_{1} \end{array}\geq \;0 \end{array}$$

In this case, tetrachoric correlations among the attributes were approximately 0.6.

**Figure 1:**
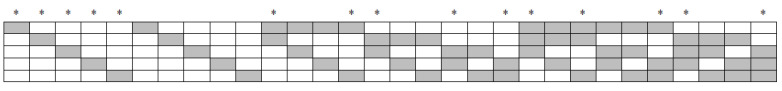
*K*-by-*I* Q’ matrix for simulation Study 1. Note: blank means ‘0,’ and gray means ‘1’; ‘*’ denotes items used in the *I* = 15 conditions.

Five attributes (*K* = 5) were measured, and the simulated Q-matrices are presented in Figure 1. Each Q-matrix contained at least one identity matrix. Each attribute was measured at least three times, which satisfies the conditions for generic identifiability (i.e., conditions C5 and C6 in Theorem 7) described by Gu and Xu (2019). Finally, the observed responses were generated from *y_ni_*~Bernoulli (*p_ni_*), where *p_ni_* was given in Equation (8) in the main text.

#### 4.1.2. Analysis

Thirty replications were implemented in each simulated condition. Two Markov chains with random starting points were used for each replication, and 10,000 iterations were run for each chain. The first 5000 iterations in each chain were discarded as burn-in. The remaining 10,000 iterations (5000 in each chain) were retained for model parameter inferences. The potential scale reduction factor (PSRF; Brooks and Gelman 1998) was computed to assess the convergence of each parameter. In this study, the PSRFs were generally less than 1.01, suggesting good convergence in the specified settings.

To evaluate parameter recovery, the bias and root mean square error (RMSE) of the item parameter estimates were computed as bias(x)=∑r=130x^r−xr30 and RMSE(x)=∑r=130( x^r−xr)230, where $\hat{x}$ and *x* were the estimated and true values of the model parameters in *r*-th replication. The attribute correct classification rate (ACCR) and attribute pattern correct classification rate (PCCR) were computed to evaluate classification accuracy, as ACCRk=∑r=130∑n=1NI(α^nkr=αnkr)NR and PCCR=∑r=130∑n=1NI(α^nr=αnr)NR.

#### 4.1.3. Results

**Figure 2:**
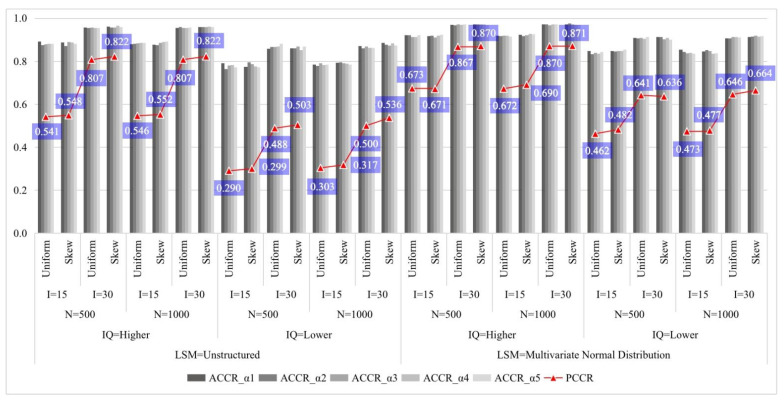
Summary of the recovery of attributes in simulation Study 1. Note: *LSM* = latent structural model; IQ = item quality; N = sample size; I = test length; ACCR = attribute correct classification rate; PCCR = attribute pattern correct classification rate.

Figure 2 summarizes the recovery of attributes (details can be found in Table S1 in the Supplementary). Referencing previous studies of CDMs with unstructured *LSM* (e.g., Ma et al. 2016; Zhan et al. 2019b), the classification accuracy of the DINMix model under different conditions is adequate and consistent with expectations. Furthermore, increasing the test length and item quality yielded higher classification accuracy. Higher correlations among attributes (i.e., when attributes were simulated from a multivariate normal distribution) led to higher classification accuracy. The sample size had a limited effect on classification accuracy. The classification accuracy in conditions with the skew type of an item-level mixing proportion seems slightly better than in conditions with the uniform type of item-level mixing proportion.

**Figure 3:**
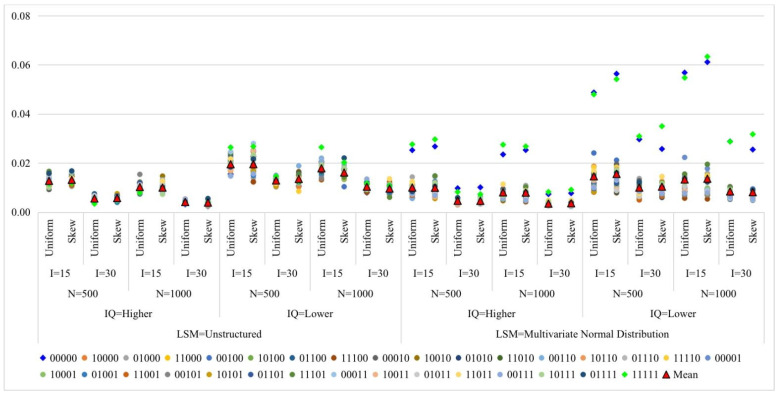
Root mean square error of attribute profile proportions in simulation Study 1. Note: *LSM* = latent structural model; IQ = item quality; N = sample size; I = test length; Mean = mean of the root mean square errors of 32 attribute profiles.

Figure 3 presents the RMSE of attribute profile proportions (details in Table S2 in the Supplementary). Except for two profiles, (00000) and (11111), which have relatively lower recovery in the multivariate normal distribution-based *LSM*, the recovery of the remaining 30 patterns is basically the same in the two *LSM*s. The main reason is that the number of people with those two extreme profiles is inherently small in the multivariate normal distribution-based *LSM*. In addition, increasing the test length and item quality yielded a smaller RSME. The sample size had a limited effect. The deviance of RMSEs in conditions with the skew type of an item-level mixing proportion appears slightly higher than in conditions with the uniform type of item-level proportion for conditions with lower item quality. 

Table 2 summarizes the recovery of item parameters. It should be noted that only their recovery in the multidimensional items (i.e., items 6–15 under I = 15 conditions and items 11–30 under I = 30 conditions) was computed for item-level mixing proportion parameters. The recovery of guessing and slip parameters was better than that of item-level mixing proportion parameters across all conditions. Larger sample sizes, longer test lengths, and higher item quality led to better item parameter recovery. Guessing and slip parameters were well recovered across different conditions. Specifically, the recovery of guessing and slip parameters under conditions with the skew type of item-level mixing proportion seems better than under conditions with the uniform type of item-level mixing proportion. Conversely, the recovery of item-level mixing proportion parameters under conditions with the skew type of item-level mixing proportion was worse than under conditions with the uniform type of item-level mixing proportion, especially for lower-quality items. Generally, the recovery of **τ**_3_ was worse than that of **τ**_1_ and **τ**_2_, mainly because τ*_i_*_3_ = 1 − (τ*_i_*_1_ + τ*_i_*_2_), and thus, τ*_i_*_3_ needs to offset both estimation errors of τ*_i_*_1_ and τ*_i_*_2_.

Overall, the results of simulation Study 1 indicate that model parameters for the DINMix model can be well recovered, as the proposed model can correctly identify items that contain coexisting condensation rules via the proposed Bayesian MCMC estimation method, especially in conditions with a larger sample, longer test length, and higher item quality.

### 4.2. Study 2

#### 4.2.1. Design and Data Generation

In simulation Study 2, six test situations were simulated. Precisely, including the situations in which the relationships between item responses and required attributes (a) follow the conjunctive condensation rule (i.e., the DINA model was used to generate data), (b) follow the disjunctive condensation rule (i.e., the DINO model was used to generate data), (c) follow the ratio condensation rule (i.e., the DINR model was used to generate data), (d) follow the compensatory condensation rule (i.e., the ACDM was used to generate data), (e) fuzzily follow some unclear condensation rules (i.e., the GDINA model was used to generate data), and (f) separately follow some clear condensation rules (i.e., five separate CDMs were combined to generate data, including DINA, DINO, DINR, ACDM, and GDINA models). Note that all the above situations are unfair to the DINMix model because it is not used as the data generation model in any of them.

**Figure 4:**
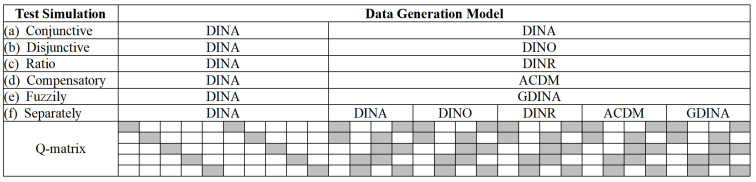
*K*-by-*I* Q’ matrix for simulation Study 2 and the data generation model for each item. Note: blank means ‘0’ and gray means ‘1’; DINA = deterministic input, noisy ‘and’ gate model; DINO = deterministic input, noisy ‘or’ gate model; DINR = deterministic input, noisy ratio model; ACDM = additive cognitive diagnosis model; GDINA = generalized DINA model; DINMix = deterministic input, noisy mixed model.

Other factors were set as control variables. More specifically, the number of attributes, sample size, and test length were fixed at 5, 1000, and 30, respectively. The Q-matrix and corresponding allocation plan of the data generation model are presented in Figure 4. The Q-matrix contained two identity matrices, and each attribute was measured at least three times to make all the mentioned CDMs identifiable (Gu and Xu 2019, 2020). In all simulated test situations, since the first 10 items were unidimensional (i.e., all CDMs were equivalent), the DINA model was forcibly assigned to them for simplicity. In the simulated test situation (f), for items 11–14, the DINA model was used as the true model; for items 15–18, the DINO model was used as the true model; for items 19–22, the DINR model was used as the true model; for items 23–26, the ACDM was used as the true model; for items 27–30, the GDINA model was used as the true model. To ensure that data were only determined by the data generation model, items with the same required attributes were assigned to each model, and all item parameters were set to fixed values.

The upper and lower limits of correct response probability were set as 0.9 and 0.1, respectively. Thus, in the DINA, DINO, and DINR models, *g_i_* and *s_i_* were both fixed at 0.1. In the ACDM, the intercept parameters were all fixed at 0.1; for two-dimensional items, two main effects were fixed at 0.5 and 0.3; for three-dimensional items, three main effects were fixed at 0.35, 0.25, and 0.2. In the GDINA model, the intercept parameters were all fixed at 0.1; for two-dimensional items, two main effects, and one two-way interaction effects were fixed at 0.35, 0.25, and 0.2, respectively; for three-dimensional items, three main effects, three two-way interaction effects, and one three-way interaction effects were fixed at 0.15, 0.1, 0.05, 0.05, 0.1, 0.15, and 0.2, respectively. The unstructured latent structural model was used (i.e., the true attribute profile of each person was randomly chosen from all possible patterns with equal probability). Finally, the observed responses of each item were generated from the corresponding model presented in Figure 1. Thirty datasets were generated in each condition.

#### 4.2.2. Analysis

In the simulated test situations (a) to (e), only the true model and the DINMix model were used to fit the generated data; while in the simulated test situation (f), five models were used to fit the generated data: DINA, DINO, DINR, DINMix, and GDINA models. All models were estimated using the Bayesian MCMC method. Analysis processes were identical to those used in simulation Study 1 (see Section S1 in the Supplementary). In addition to bias, the RMSE, the PCCR, the deviance information criterion (DIC; Spiegelhalter et al. 2002), and the log conditional predictive ordinate (Kim and Bolt 2007; Levy and Mislevy 2016, p. 247) multiplied by –2 (i.e., –2LCPO) were computed for model selection. Both the test- and item-level –2LCPO were reported. A smaller value of the DIC and –2LCPO indicates a better model-data fit.

#### 4.2.3. Results

Table 3 presents the overall performance of six models in simulation Study 2, including model-data fitting, the recovery of item parameters, the recovery of attributes, and the recovery of attribute profile proportions (details can be found in Table S2 and Figure S1 in Supplementary). For the first five test situations, it was unsurprising that the overall performance of the DINMix model was worse than that of the data generation model itself, but the relative disadvantage of the former was minimal, even in these extremely unfair test situations. Additionally, both model-data fitting indicators can successfully identify the true model, indicating that the degree of model-data fitting can be used as evidence of the validity of the measurement of cognitive processes. For the last test situation, the overall performance of the six models was evaluated in the following relative order: GDINA ≥ DINMix >> ACDM ≥ DINR > DINA > DINO. More specifically, first, the GDINA model performs best in complex test situations, as expected, because it is a saturated model; second, the performance of the DINMix model was slightly worse than that of the GDINA model; third, the performance of the first two models was much better than that of the last four reduced models; fourth, the ACDM performed only slightly better than the DINR model.

**Figure 5:**
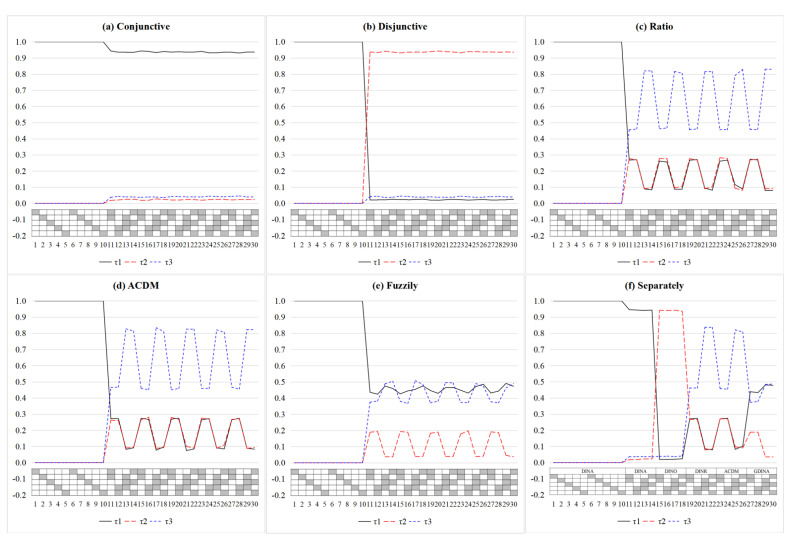
Estimates of the item-level mixing proportion parameter in simulation Study 2 from the DINMix model. Note: The value is the mean value of 30 replications; τ_1_ = item-level mixing proportion parameter for the conjunctive condensation rule; τ_2_ = item-level mixing proportion parameter for the disjunctive condensation rule; τ_3_ = item-level mixing proportion parameter for the ratio condensation rule; DINMix = deterministic input, noisy mixed model; GDINA = generalized DINA model.

Figure 5 presents the estimates of item-level mixing proportion parameters of the DINMix model in six test situations. First, by using the estimate of τ*_i_*_1_ > 0.9 and τ*_i_*_2_ > 0.9 as a judgment condition, τ*_i_*_1_ and τ*_i_*_2_ can accurately identify the conjunctive and disjunctive condensation rules for each item across all test situations, respectively. For τ*_i_*_3_, we cannot make a judgment directly by using a certain cut-point (e.g., 0.9), as for τ*_i_*_1_ and τ*_i_*_2_; however, by judging whether τ*_i_*_3_ is simultaneously larger than τ*_i_*_1_ and τ*_i_*_2_, the items that follow the ratio/compensatory condensation rule can still be identified. Second, when the relationships between item responses and required attributes fuzzily followed some condensation rules (i.e., the test situation (e)), τ*_i_*_1_, τ*_i_*_2_, and τ*_i_*_3_ seemed to show the following pattern: the proportion of τ*_i_*_1_ and τ*_i_*_3_ was much higher than that of τ*_i_*_2_. Of course, this pattern may change depending on the simulated values of different item parameters in the GDINA model. Third, for test situation (f), even in such a complex test situation, the DINMix model can identify the condensation rules followed by each item by adaptively adjusting the estimates of τ*_i_*_1_, τ*_i_*_2_, and τ*_i_*_3_. If the identified condensation rules for some items were inconsistent with those predefined by experts, then the experts may consider revising these items.

**Figure 6:**
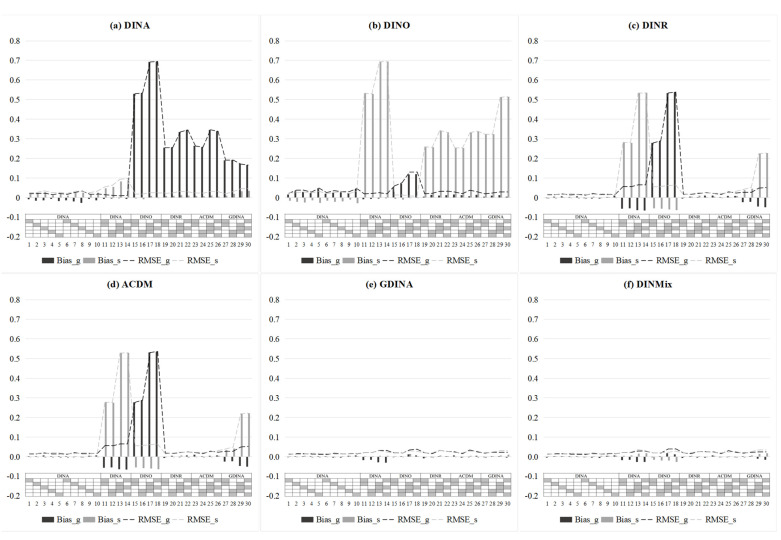
Summary of the recovery of item parameters in simulation Study 2. Note, RMSE = root mean square error; DINA = deterministic input, noisy ‘and’ gate model; DINO = deterministic input, noisy ‘or’ gate model; DINR = deterministic input, noisy ratio model; ACDM = additive cognitive diagnosis model; GDINA = generalized DINA model; DINMix = deterministic input, noisy mixed model.

To better illustrate the comparative advantages and disadvantages of the proposed model, the following discussion focuses on test situation (f). Figure 6 summarizes the recovery of the item parameters of six models (details can be found in Table S3 in the Supplementary). Only the recoveries of the upper and lower limits of the correct response probability (i.e., the guessing and slip parameters) were computed. The DINMix and GDINA models performed well in recovering the parameters in all items and were unaffected by the true condensation rule followed by each item. Additionally, when the true condensation rule followed by an item did not match the condensation rule adopted by the analysis model, the DINA model substantially overestimated the guessing parameter, while the DINO model substantially overestimated slip parameters, and the DINR model’s results were a bit more complicated. Specifically, when the true condensation rule was conjunctive, the DINR model substantially overestimated slip parameters and slightly underestimated guessing parameters; however, when the true condensation rule was disjunctive, the DINR model substantially overestimated guessing parameters and slightly underestimated slip parameters. Furthermore, the ACDM still performed similarly to the DINR model.

**Figure 7:**
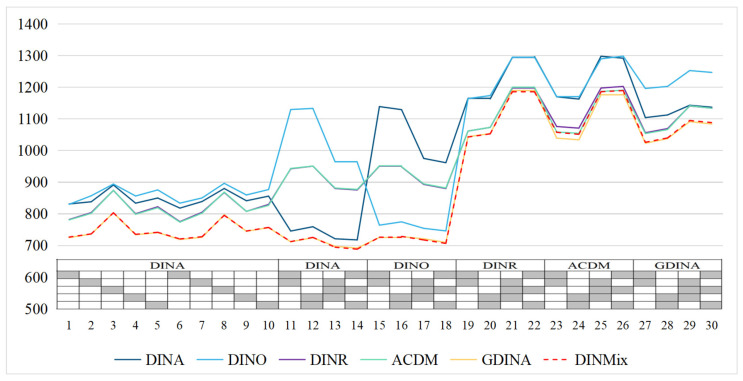
Item-level –2LCPO of six models in simulation Study 2. Note: The value is the mean value of 30 replications; –2LCPO = –2 log conditional predictive ordinate; DINA = deterministic input, noisy ‘and’ gate model; DINO = deterministic input, noisy ‘or’ gate model; DINR = deterministic input, noisy ratio model; ACDM = additive cognitive diagnosis model; GDINA = generalized DINA model; DINMix = deterministic input, noisy mixed model.

Figure 7 displays the item-level –2LCPO of the six models (details in Table S4 in the Supplementary). The GDINA and the DINMix models were more effective than the other four reduced models in fitting the data on most, if not all, items, regardless of the true model for each item. The DINA, DINO, DINR, and ACDM models had a relatively high degree of item-level fit, but only when their adopted condensation rule conformed to the true condensation rule followed by an item; in contrast, the GDINA and DINMix models seemed to be unaffected by the true condensation rule followed by the item.

Overall, despite these unfair test situations, the DINMix model performed well. The results of simulation Study 2 indicate that (a) the DINMix model can adaptively identify different condensation rules existing separately in multiple items, and (b) the overall performance of the DINMix model is similar to that of the GDINA model, both of which are suitable for complex test situations. However, it should be noted that the DINMix and GDINA models deal with complex test situations differently. The former identifies condensation rules followed by different items by adjusting the item-level mixing proportion parameters, while the latter accounts for all possible condensation rules through saturated interaction effects. Thus, the analysis results of the former are more helpful for item revision from the perspective of improving validity.

## 5. An Empirical Example

A commonly used empirical dataset was used to illustrate the applicability and advantages of the proposed model: the fraction subtraction (FS) dataset (Tatsuoka 2002). Since this dataset has been widely used in many previous studies, it does not need to be described in detail here. In addition to the DINA, DINO, DINR, ACDM, GDINA, and DINMix models, a selected mixing model via the Wald test (Ma et al. 2016) was used to fit the data. The analysis processes for the first six models were identical to those used in simulation studies. To make the results comparable, the Wald test was first used for the selected mixing model to select a suitable reduced CDM for each item using the GDINA package (Ma and de la Torre 2020) in R software. The candidate-reduced models included the DINA, DINO, and ACDM. Then, according to the selected models^3^, Bayesian estimation was used. The DIC and –2LCPO were computed for model selection.

**Figure 8:**
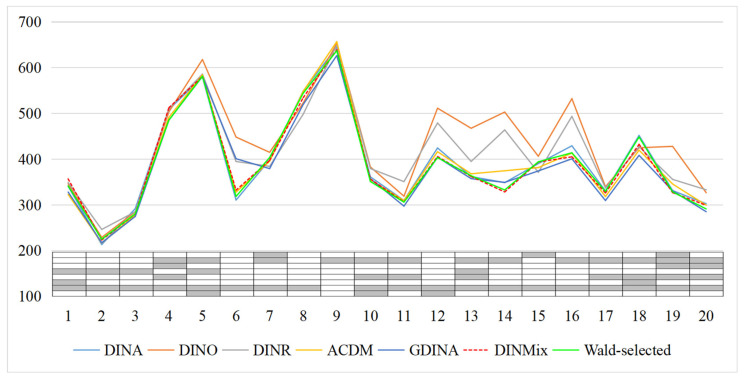
Item-level –2LCPO of seven models for the fraction subtraction data. Note: DINA = deterministic input, noisy ‘and’ gate model; DINO = deterministic input, noisy ‘or’ gate model; DINR = deterministic input, noisy ratio model; ACDM = additive cognitive diagnosis model; GDINA = generalized DINA model; DINMix = deterministic inputs, noisy mixed model; Wald-selected = selected mixing model via Wald test.

The FS dataset consists of responses given by 536 individuals to 20 items measuring eight attributes. The Q-matrix was published by Tatsuoka (2002)^4^. Previous studies have shown that the DINA model can fit these data well (e.g., DeCarlo 2011; de la Torre and Douglas 2004). Table 4 presents the DIC and test-level –2LCPO of seven models. The DINMix model was preferred based on the DIC, and the GDINA model was selected based on the test-level –2LCPO. Additionally, Figure 8 displays the item-level –2LCPO of the seven models. The DINA, DINMix, GDINA, and selected mixing models have approximate fittings, mainly because the latter three also agree that the conjunctive condensation rule is more suitable for most items.

**Figure 9:**
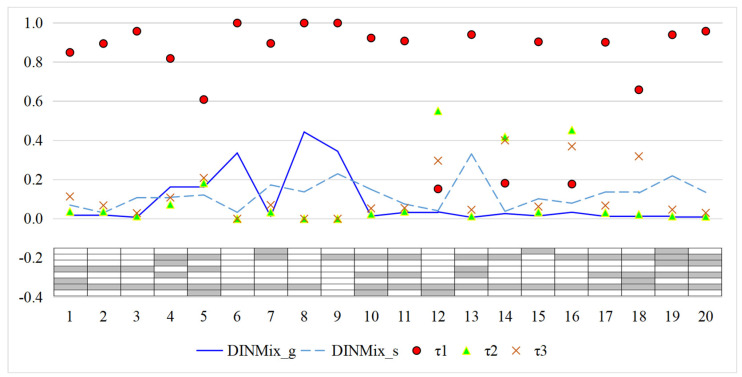
The estimated item parameters for the fraction subtraction data from the DINMix models. Note: g = guessing parameter; s = slip parameter; τ_1_ = item-level mixing proportion parameter for the conjunctive condensation rule; τ_2_ = item-level mixing proportion parameter for the disjunctive condensation rule; τ_3_ = item-level mixing proportion parameter for the ratio condensation rule; DINMix = deterministic input, noisy mixed model.

Figure 9 presents the estimated item parameters of the DINMix model for the FS data (the results of other models are provided in Tables S5 and S6 in the Supplementary). The estimates of τ*_im_*s indicate that the conjunctive condensation rule accounts for the largest proportion of most items. When using the judgment rules obtained in simulation Study 2 (e.g., τ*_i_*_1_ > 0.9 or τ*_i_*_2_ > 0.9), it was difficult to determine which specific condensation rule that items 1, 4, 5, 12, 14, 16, and 18 followed, primarily because they were judged to contain coexisting condensation rules. Similarly, the results of the Wald test also suggest that no particular condensation rule applied to items 1, 4, 12, 14, and 16. This consistency also indicates that the proposed model can further explain why the Wald test cannot find a specific condensation rule for some items. Additionally, use the item 14, $3\frac{4}{5}-3\frac{2}{5}$,^5^ as an example. Attributes α_2_ (separate a whole number from a fraction) and α_7_ (subtract numerators) were required to respond correctly according to the Q-matrix. However, respondents who mastered α_7_ but not α_2_ could still identify that the correct answer was 2/5. The first reason is that respondents may ignore the integer part and only focus on the difference between the fraction part. The second reason is that alternative attributes that are unspecified by the Q-matrix can probably be used to answer this item, such as *convert mixed number to fraction* (Mislevy 1996). Apparently, for whatever reason, the expert-defined conjunctive condensation rule does not fully apply to this item, which is also what the DINMix model indicated.

## 6. Summary and Discussion

The condensation rule describes the logical relationship between the required attributes and the item response, reflecting the explicit assumption about respondents’ cognitive processes to solve problems. When an item contains coexisting condensation rules, the contribution of required attributes to the correct item response probability follows multiple condensation rules with different proportions. Coexisting condensation rules reflect the complexity of cognitive processes in problem solving and that the cognitive processes respondents employ in their item responses are inconsistent with the expert-designed condensation rule. This study proposed the DINMix model to identify coexisting condensation rules to provide feedback for item revision. Two simulation studies were conducted to evaluate the psychometric properties of the proposed model. The simulation results indicate that (a) the model parameters for the DINMix model can be well recovered; (b) the DINMix model can adaptively and accurately identify coexisting condensation rules, either existing simultaneously in an item or existing separately in multiple items; (c) the overall performance of the DINMix model is similar to that of the GDINA model, both of which are suitable for complex test situations. An empirical example was also analyzed to illustrate the applicability and advantages of the proposed model.

The work represented in this article is an initial attempt to consider multiple condensation rules in a single CDM simultaneously. Despite the promising results, some limitations remain. First, the utilized model framework (see Equations (1) and (5)) models aberrant responses at the item level. However, in practice, such unusual responses may occur at the attribute rather than the item level, such as noisy input, deterministic, and gate model (Junker and Sijtsma 2001). Exploring ways to incorporate attribute-level aberrant responses into the proposed model is worthy of further research, for which Equation (11) of de la Torre (2011) seems to give us a reference. Second, within-item characteristic dependency (Zhan et al. 2019b) was not considered in the proposed model, which means that dependency exists between the guessing and slip parameters within an item. It can be incorporated into the proposed model to increase the estimation accuracy of item parameters in a future study. Third, only the dichotomous scoring item and binary attribute were modeled in the proposed model. Extending the current model to consider polytomous scoring items (e.g., Ma and de la Torre 2016) and polytomous attributes (e.g., Zhan et al. 2020) would be meaningful and practical. Fourth, in recent years, some studies have focused on Q-matrix validation or estimation (Chen et al. 2018; de la Torre and Chiu 2016) and multiple strategies for problem solving (Ma and Guo 2019), which are not covered in the current study. Fifth, inspired by the modeling logic of mixture item response models, this study proposed the idea of the coexistence of multiple factors (e.g., condensation rules) at the item level. This item-level mixed-modeling idea may have some extended uses worth exploring, such as coexisting problem-solving strategies and coexisting Q-matrices (i.e., q-vectors, to be exact) at the item level. Sixth, notably, the generalizability of the findings of this study is dependent upon the limitations of the design of the simulation studies, such as using a fixed number of attributes and assuming the Q-matrix is correct. To further generalize these findings, a wider range of simulated conditions should be considered in future studies. Seventh, in addition to the MCMC algorithm used in this study, subsequent attempts can be made to use the maximum likelihood estimation with a potentially shorter computing time. Eighth, 30 sets of data were randomly generated in the simulation study, which may not be sufficient to eliminate the impact of random error.

Lastly, model identifiability is essential for valid statistical inferences, but determining the identifiability conditions could be challenging. Although the conditions for generic identifiability for the multi-parameter Q-restricted latent class model are also applicable to the DINMix model, it remains to be explored whether there are other specific identifiable requirements for the DINMix model. In other words, the identifiability conditions of the proposed model have yet to be established. In cognitive diagnosis, many CDMs were proposed without addressing the issue of model identifiability, and researchers have long recognized that CDMs are generally not identifiable (DeCarlo 2011; DiBello et al. 1995). For example, even the identifiability condition of the most practiced DINA model was not solved until more than 10 years after it was proposed (Xu and Zhang 2016; Xu 2017). Similarly, the identifiability condition of the GDINA model was only recently addressed (Gu and Xu 2020). Although the unified model (DiBello et al. 1995) also had an unidentifiable issue when it was proposed, it will be solved as the research advances (Hartz and Roussos 2008). In addition to those early proposed models, some recent new CDMs with identifiability issues still need to be addressed (e.g., Ma 2022).

## Figures and Tables

**Table 1:** The Correct Response Probabilities of Four Deterministic Input, Noisy Models for an Item.

Attribute Profile	Number of Mastered Attributes	DINA	DINO	DINR	DINMix
(0, 0, 0)	0	0.1	0.1	0.1	0.1
(1, 0, 0)	1	0.1	0.9	0.367	0.207
(0, 1, 0)	1	0.1	0.9	0.367	0.207
(0, 0, 1)	1	0.1	0.9	0.367	0.207
(1, 1, 0)	2	0.1	0.9	0.633	0.233
(1, 0, 1)	2	0.1	0.9	0.633	0.233
(0, 1, 1)	2	0.1	0.9	0.633	0.233
(1, 1, 1)	3	0.9	0.9	0.9	0.9

Note: Item characteristics are **q***_i_* = (1, 1, 1), *g_i_* = *s_i_* = 0.1, and τ*_i_*_1_ = 0.8, τ*_i_*_2_ = 0.1, where **q***_i_* is the required attribute profile of item *i*, *g_i_*, and *s_i_* is the guessing and slip parameters of item *i*, respectively; τ*_i_*_1_ is the item-level mixing proportion parameter for the conjunctive condensation rule; τ*_i_*_2_ is the item-level mixing proportion parameter for the disjunctive condensation rule; DINA = deterministic input, noisy ‘and’ gate model; DINO = deterministic input, noisy ‘or’ gate model; DINR = deterministic input, noisy ratio model; DINMix = deterministic input, noisy mixed model.

**Table 2:** Summary of the Recovery of Item Parameters in Simulation Study 1.

*LSM*	*IQ*	*N*	*I*	*TM*	*g*		*s*		τ_1_		τ_2_		τ_3_	
					Bias	RMSE	Bias	RMSE	Bias	RMSE	Bias	RMSE	Bias	RMSE
Un	High	500	15	Unif	0.017	0.048	0.020	0.054	−0.041	0.100	−0.048	0.107	0.089	0.150
				Skew	0.015	0.049	0.020	0.049	0.039	0.089	0.023	0.107	−0.061	0.154
			30	Unif	0.013	0.037	0.013	0.035	−0.036	0.083	−0.041	0.090	0.077	0.121
				Skew	0.009	0.036	0.011	0.035	−0.023	0.089	−0.013	0.084	0.037	0.134
		1000	15	Unif	0.009	0.040	0.014	0.038	−0.029	0.085	−0.024	0.093	0.053	0.132
				Skew	0.008	0.036	0.007	0.036	0.013	0.076	0.013	0.087	−0.026	0.130
			30	Unif	0.005	0.025	0.008	0.026	−0.042	0.078	−0.035	0.075	0.077	0.126
				Skew	0.006	0.024	0.007	0.024	−0.025	0.073	−0.026	0.072	0.050	0.119
	Low	500	15	Unif	0.012	0.059	0.022	0.065	−0.019	0.080	−0.020	0.085	0.039	0.077
				Skew	0.013	0.063	0.018	0.064	0.084	0.117	0.054	0.153	−0.138	0.215
			30	Unif	0.011	0.049	0.017	0.048	−0.030	0.096	−0.015	0.099	0.045	0.081
				Skew	0.008	0.050	0.012	0.047	−0.009	0.121	0.003	0.124	0.006	0.167
		1000	15	Unif	0.006	0.048	0.015	0.055	−0.026	0.099	−0.012	0.097	0.039	0.087
				Skew	0.001	0.052	0.010	0.049	0.081	0.131	0.036	0.144	−0.117	0.200
			30	Unif	0.006	0.037	0.010	0.039	−0.034	0.092	−0.031	0.094	0.065	0.104
				Skew	0.001	0.036	0.008	0.036	−0.013	0.099	−0.009	0.107	0.022	0.145
MVN	High	500	15	Unif	0.007	0.031	0.007	0.031	−0.039	0.089	−0.039	0.090	0.078	0.130
				Skew	0.009	0.032	0.006	0.030	0.052	0.104	0.036	0.113	−0.089	0.173
			30	Unif	0.005	0.026	0.004	0.025	−0.043	0.086	−0.039	0.086	0.083	0.125
				Skew	0.005	0.026	0.005	0.026	−0.017	0.095	−0.014	0.089	0.031	0.147
		1000	15	Unif	0.004	0.023	0.005	0.023	−0.037	0.087	−0.038	0.093	0.076	0.156
				Skew	0.002	0.021	0.006	0.022	0.019	0.085	0.027	0.094	−0.046	0.146
			30	Unif	0.003	0.018	0.004	0.018	−0.040	0.079	−0.044	0.080	0.084	0.133
				Skew	0.002	0.018	0.003	0.018	−0.019	0.077	−0.019	0.078	0.037	0.132
	Low	500	15	Unif	−0.001	0.040	0.002	0.041	−0.032	0.097	−0.036	0.097	0.069	0.095
				Skew	−0.004	0.041	−0.001	0.039	0.087	0.134	0.041	0.141	−0.128	0.201
			30	Unif	0.004	0.034	0.007	0.034	−0.033	0.096	−0.028	0.097	0.061	0.090
				Skew	0.002	0.034	0.006	0.033	−0.008	0.126	0.001	0.124	0.007	0.167
		1000	15	Unif	0.000	0.031	0.001	0.029	−0.040	0.105	−0.029	0.103	0.069	0.110
				Skew	−0.003	0.031	0.004	0.031	0.057	0.109	0.044	0.127	−0.102	0.176
			30	Unif	0.001	0.025	0.005	0.024	−0.037	0.096	−0.033	0.092	0.071	0.106
				Skew	0.001	0.023	0.005	0.023	−0.011	0.099	−0.007	0.098	0.018	0.149

Note, *g* = guessing parameter; *s* = slip parameter; τ_1_ = item-level mixing proportion parameter for the conjunctive condensation rule; τ_2_ = item-level mixing proportion parameter for the disjunctive condensation rule; τ_3_ = item-level mixing proportion parameter for the ratio condensation rule; *LSM* = latent structural model; *IQ* = item quality; *N* = sample size; *I* = test length; *TM* = type of item-level mixing proportion; Un = unstructured *LSM*; MVN = multivariate normal distribution; Unif = uniform; RMSE = root mean square error.

**Table 3:** Summary of the Results of Simulation Study 2.

Test Situation	Analysis Model	DIC	Test_–2LCPO	RMSE_g	RMSE_s	PCCR	RMSE_α
Conjunctive	DINA	20,748.69	21,201.02	0.012	0.021	0.805	0.006
	DINMix	20,852.34	21,256.41	0.024	0.022	0.804	0.006
Disjunctive	DINO	20,746.62	21,200.64	0.022	0.013	0.806	0.006
	DINMix	20,827.94	21,251.88	0.022	0.024	0.803	0.006
Ratio	DINR	29,963.85	30,625.57	0.020	0.020	0.853	0.004
	DINMix	30,020.02	30,676.92	0.021	0.021	0.852	0.004
Compensatory	ACDM	29,613.86	30,390.21	0.020	0.020	0.853	0.003
	DINMix	30,084.68	30,713.51	0.022	0.021	0.844	0.004
Fuzzily	GDINA	28,541.86	29,260.53	0.020	0.022	0.845	0.004
	DINMix	28,743.04	29,372.46	0.023	0.024	0.840	0.004
Separately	DINA	29,348.12	29,971.11	0.195	0.035	0.746	0.007
	DINO	30,078.30	30,616.86	0.039	0.227	0.726	0.008
	DINR	28,386.28	28,972.58	0.079	0.093	0.827	0.004
	ACDM	28,266.83	28,911.75	0.079	0.092	0.828	0.004
	GDINA	25,595.61	26,341.86	0.022	0.022	0.900	0.003
	DINMix	25,744.26	26,397.83	0.021	0.022	0.899	0.003

Note: The value is the mean value of 30 replications; DIC = deviance information criterion; Test_–2LCPO = test-level –2 log conditional predictive ordinate; RMSE_g = mean root mean square errors of guessing parameter across all items; RMSE_s = mean RMSEs of slipping parameters across all items; RMSE_α = mean RMSEs of 32 attribute profile proportions; PCCR = attribute pattern correct classification rate; DINA = deterministic input, noisy ‘and’ gate model; DINO = deterministic input, noisy ‘or’ gate model; DINR = deterministic input, noisy ratio model; ACDM = additive cognitive diagnosis model; GDINA = generalized DINA model; DINMix = deterministic input, noisy mixed model.

**Table 4:** The DIC and –2LCPO of Seven Models for the Fraction Subtraction Data.

Analysis Model	DIC	Test-Level –2LCPO
DINA	8347.341	7882.304
DINO	9038.505	8665.394
DINR	8911.684	8293.461
ACDM	10,528.705	7826.567
GDINA	11,096.800	7690.248
DINMix	8330.826	7803.378
Wald-selected	8785.672	7783.750

Note: DIC = deviance information criterion; –2LCPO = –2 log conditional predictive ordinate; DINA = deterministic input, noisy ‘and’ gate model; DINO = deterministic input, noisy ‘or’ gate model; DINR = deterministic input, noisy ratio model; ACDM = additive cognitive diagnosis model; GDINA = generalized DINA model; DINMix = deterministic input, noisy mixed model; Wald-selected = selected mixing model via Wald test.

## Data Availability

The toy data and running code for the models used in this study are available at https://osf.io/s2yjv/.

## Notes

1 Specifically, it follows the data-first philosophy (i.e., by developing more complex models to realize the analysis of data in complex situations). In contrast, for the perspective of measurement, considering the viewpoint of garbage-in, garbage-out for predetermined measurement objects (e.g., attributes and cognitive processes) to ensure the reliability and validity of measurement, factors unrelated to the measurement should be excluded (i.e., be revised or be deleted) as much as possible.

2 Any one element in **τ***_i_* can be set to 1 because all CDMs (for binary attributes) are identical to each other for unidimensional items.

3 According to the *p*-values of the Wald test, items 1, 4, 12, 14, and 16 are applicable to the GDINA model, while the remaining items are applicable to the DINA model.

4 The author does, however, note that this Q-matrix has been suggested to be revised by some previous studies (e.g., Chen et al. 2018). However, it is still fair to compare several models with the same Q-matrix, especially when there is no definite conclusion about the revision of this Q-matrix.

5 The specific content of each item can be found in table 1 in DeCarlo (2011).

## Supplementary Materials

The following supporting information can be downloaded at: https://www.mdpi.com/article/10.3390/jintelligence11030055/s1, Section S1. The relationship between DINMix model and GDINA model; Figure S1. Root Mean Square Error of Attribute Profile Proportions in Simulation Study 2; Table S1. Summary of the Recovery of Attributes in Simulation Study 1; Table S2. Summary of the Recovery of Attributes in Simulation Study 2; Table S3. Summary of the Recovery of Item Parameters in Test Situation (f) in Simulation Study 2; Table S4. Summary of the Item-Level –2LCPO of Six Models in Test Situation (f) in Simulation Study 2; Table S5. The Estimated Item Parameters for the Fraction Subtraction Data (Posterior Mean); Table S6. The Estimated Item Parameters for the Fraction Subtraction Data (Posterior Standard Deviation).
