# Supplementary material for: Deterministic Input, Noisy Mixed Modeling for Identifying Coexisting Condensation Rules in Cognitive Diagnostic Assessments
Source: J Intell. 2023 Mar 16;11(3):55. doi: 10.3390/jintelligence11030055 (PMC10056588; doi:10.3390/jintelligence11030055)
Supplement: Supplementary file 1 [file jintelligence-11-00055-s001.zip › jintelligence-2127567-supplementary.pdf]

Supplementary

Section S1. The relationship between DINMix model and GDINA model

To understand the relationship between DINMix model and GDINA model, for simplicity, let us take an item with two required attributes as an example (i.e., $\mathbf{q}_i$ = (1, 1)). In such case, the GDINA model can be expressed as

$$P(y_{ni}=1|\boldsymbol{\delta}_i,\alpha_{nk})=\delta_{i0}+\delta_{i1}\alpha_{n1}+\delta_{i2}\alpha_{n2}+\delta_{i12}\alpha_{n1}\alpha_{n2}, \quad \text{(S1)}$$

where δ_{i0} is the intercept for item i; δ_{i1} and δ_{i2} are the main effect due to α_1 and α_2, respectively; δ_{i12} is the interaction effect due α_1 and α_2. In contrast, for the same item, the DINMix model can be expressed as

$$\begin{aligned}
P(y_{ni}=1|g_i,s_i,\boldsymbol{\tau}_i,\alpha_{nk}) &= g_i+(1-s_i-g_i)\left(\tau_{i1}\alpha_{n1}\alpha_{n2}+\tau_{i2}\left(1-(1-\alpha_{n1})(1-\alpha_{n2})\right)+\tau_{i3}\frac{\alpha_{n1}+\alpha_{n2}}{2}\right)\\
&= g_i+(1-s_i-g_i)\left(\tau_{i1}\alpha_{n1}\alpha_{n2}+\tau_{i2}\alpha_{n1}+\tau_{i2}\alpha_{n2}-\tau_{i2}\alpha_{n1}\alpha_{n2}+(1-\tau_{i1}-\tau_{i2})\frac{(\alpha_{n1}+\alpha_{n2})}{2}\right)\\
&= g_i+(1-s_i-g_i)\left(\frac{(1-\tau_{i1}+\tau_{i2})}{2}\alpha_{n1}+\frac{(1-\tau_{i1}+\tau_{i2})}{2}\alpha_{n2}+(\tau_{i1}-\tau_{i2})\alpha_{n1}\alpha_{n2}\right)\\
&= g_i+\frac{(1-s_i-g_i)(1-\tau_{i1}+\tau_{i2})}{2}\alpha_{n1}+\frac{(1-s_i-g_i)(1-\tau_{i1}+\tau_{i2})}{2}\alpha_{n2}+(1-s_i-g_i)(\tau_{i1}-\tau_{i2})\alpha_{n1}\alpha_{n2}
\end{aligned} . \quad \text{(S2)}$$

Comparing equations (S1) and (S2), it can be found that the DINMix model is equal to the GDINA model by setting intercept parameter $\delta_{i0}\equiv g_i$, main effect $\delta_{i1}=\delta_{i2}\equiv\frac{(1-s_i-g_i)(1-\tau_{i1}+\tau_{i2})}{2}$, and two-way interaction effect $\delta_{i12}\equiv(1-s_i-g_i)(\tau_{i1}-\tau_{i2})$.

We can also observe the difference between above two models (S1 and S2) in how they are constrained as reduced models. For the GDINA model, it has

$$P(y_{ni}=1|\boldsymbol{\delta}_i,\alpha_{nk})=\begin{cases} DINA: & \delta_{i0}+\delta_{i12}\alpha_{n1}\alpha_{n2} \quad if \quad \delta_{i1}=\delta_{i2}=0\\ DINO: & \delta_{i0}+\delta_{i1}\alpha_{n1}+\delta_{i2}\alpha_{n2}-\delta_{i12}\alpha_{n1}\alpha_{n2} \quad if \quad \delta_{i1}=\delta_{i2}=-\delta_{i12}\\ ACDM: & \delta_{i0}+\delta_{i1}\alpha_{n1}+\delta_{i2}\alpha_{n2} \quad if \quad \delta_{i12}=0\end{cases} . \quad \text{(S3)}$$

By contrast, for the DINMix model, it has

$$P(y_{ni}=1|g_i,s_i,\boldsymbol{\tau}_i,\alpha_{nk})=\begin{cases} DINA: & g_i+(1-s_i-g_i)(\alpha_{n1}\alpha_{n2}) \quad if \quad \tau_{i1}=1\\ DINO: & g_i+(1-s_i-g_i)(\alpha_{n1}+\alpha_{n2}-\alpha_{n1}\alpha_{n2}) \quad if \quad \tau_{i2}=1\\ DINR: & g_i+(1-s_i-g_i)(\alpha_{n1}/2+\alpha_{n2}/2) \quad if \quad \tau_{i3}=1\end{cases} . \quad \text{(S4)}$$

It can be found that (a) the GDINA model achieves reduction by directly constraining the item parameters, while the DINMix model achieves reduction by constraining the item-level mixing proportions parameters; that (b) when the two models are constrained to the DINA and DINO models, there is no essential difference between them; and that (c) the main difference between them is reflected in the reduced models that satisfy the compensatory condensation rule.

Further, with the increase of attributes required by the item, the generalized relationship between the GDINA model and the DINMix model can be expressed as

$$\text{Intercept}: \delta_{i0} \equiv g_i,$$
$$\text{Main effect}: \delta_{ik} \equiv (1-s_i-g_i)\frac{1-\tau_{i1}+(K_i^*-1)\tau_{i2}}{K^*}, \quad \text{(S5)}$$
$$x\text{-way interaction effect}: \delta_{i(x)} \equiv (1-s_i-g_i)(\tau_{i1}\mathrm{I}(x=K_i^*)+\tau_{i2}(-1)^{K_i^*+1}),$$

where $K_i^* = \sum_{k=1}^{K} q_{ik}$ is the number of required attributes of item *i*; I(·) is an identification function.

Overall, the DINMix model can be seen as a constraint model from the GDINA model by fixing some parameters (e.g., main effects) of the latter to be equal.

Table S1. Summary of the Recovery of Attributes in Simulation Study 1.

LSM	*IQ*	*N*	*I*	*TM*	ACCR					PCCR
					α_1	α_2	α_3	α_4	α_5	
Un	Higher	500	15	Uniform	0.891	0.876	0.880	0.882	0.881	0.541
				Skew	0.887	0.871	0.890	0.888	0.881	0.548
			30	Uniform	0.958	0.956	0.957	0.955	0.956	0.807
				Skew	0.961	0.958	0.957	0.965	0.959	0.822
		1000	15	Uniform	0.879	0.881	0.883	0.886	0.887	0.546
				Skew	0.878	0.875	0.886	0.889	0.892	0.552
			30	Uniform	0.956	0.959	0.955	0.955	0.958	0.807
				Skew	0.959	0.960	0.960	0.962	0.960	0.822
	Lower	500	15	Uniform	0.790	0.763	0.781	0.782	0.772	0.290
				Skew	0.775	0.796	0.788	0.777	0.773	0.299
			30	Uniform	0.858	0.866	0.866	0.870	0.883	0.488
				Skew	0.861	0.861	0.869	0.852	0.869	0.503
		1000	15	Uniform	0.786	0.779	0.792	0.784	0.785	0.303
				Skew	0.794	0.796	0.792	0.790	0.786	0.317
			30	Uniform	0.871	0.861	0.870	0.863	0.862	0.500
				Skew	0.886	0.878	0.873	0.885	0.873	0.536
MVN	Higher	500	15	Uniform	0.922	0.921	0.913	0.913	0.922	0.673
				Skew	0.918	0.919	0.912	0.920	0.923	0.671
			30	Uniform	0.971	0.969	0.972	0.971	0.972	0.867
				Skew	0.972	0.972	0.972	0.973	0.969	0.870
		1000	15	Uniform	0.920	0.918	0.920	0.920	0.916	0.672
				Skew	0.923	0.918	0.922	0.928	0.926	0.690
			30	Uniform	0.972	0.971	0.968	0.973	0.973	0.870
				Skew	0.972	0.976	0.972	0.969	0.970	0.871
	Lower	500	15	Uniform	0.849	0.834	0.840	0.836	0.845	0.462
				Skew	0.848	0.846	0.848	0.848	0.855	0.482
			30	Uniform	0.910	0.907	0.909	0.905	0.913	0.641
				Skew	0.914	0.914	0.903	0.909	0.900	0.636
		1000	15	Uniform	0.854	0.844	0.837	0.840	0.835	0.473
				Skew	0.847	0.852	0.849	0.836	0.839	0.477
			30	Uniform	0.907	0.906	0.913	0.913	0.912	0.646

	Skew	0.914	0.916	0.919	0.915	0.918	0.664

Note, LSM = latent structural model; IQ = item quality; N = sample size; I = test length; TM = type of item-level mixing proportion; Un = unstructured LSM; MVN = multivariate normal distribution; ACCR = attribute correct classification rate; PCCR = pattern correct classification rate.

Table S2. Summary of the Recovery of Attributes in Simulation Study 2

Test Situation	Analysis Model	ACCR					PCCR
		α_1	α_2	α_3	α_4	α_5	
Conjunctive	DINA	0.961	0.958	0.942	0.960	0.958	0.805
	DINMix	0.960	0.958	0.941	0.960	0.958	0.804
Disjunctive	DINO	0.960	0.960	0.941	0.959	0.957	0.806
	DINMix	0.959	0.959	0.941	0.958	0.957	0.803
Ratio	DINR	0.971	0.970	0.956	0.969	0.970	0.853
	DINMix	0.971	0.970	0.955	0.968	0.969	0.852
Compensatory	ACDM	0.981	0.966	0.956	0.976	0.956	0.853
	DINMix	0.979	0.962	0.955	0.973	0.954	0.844
Fuzzily	GDINA	0.975	0.965	0.954	0.970	0.962	0.845
	DINMix	0.973	0.964	0.952	0.970	0.961	0.840
Separately	DINA	0.949	0.946	0.927	0.949	0.941	0.746
	DINO	0.947	0.939	0.923	0.945	0.935	0.726
	DINR	0.969	0.964	0.950	0.966	0.960	0.827
	ACDM	0.969	0.964	0.949	0.968	0.960	0.828
	GDINA	0.982	0.981	0.965	0.981	0.979	0.900
	DINMix	0.981	0.980	0.965	0.980	0.979	0.899

Note, ACCR = attribute correct classification rate; PCCR = attribute pattern correct classification rate; DINA = deterministic input, noisy "and" gate model; DINO = deterministic input, noisy "or" gate model; DINR = deterministic input, noisy ratio model; ACDM = additive cognitive diagnosis model; GDINA = generalized DINA model; DINMix = deterministic input, noisy mixed model.

Table S3. Summary of the Recovery of Item Parameters in Test Situation (f) in Simulation Study 2.

Item	DINA				DINO				DINR				ACDM				GDINA				DINMix			
	g_i		s_i		g_i		s_i		g_i		s_i		g_i*		s_i*		g_i*		s_i*		g_i		s_i	
	Bias	RMSE	Bias	RMSE	Bias	RMSE	Bias	RMSE	Bias	RMSE	Bias	RMSE	Bias	RMSE	Bias	RMSE	Bias	RMSE	Bias	RMSE	Bias	RMSE	Bias	RMSE
1	–0.009	0.021	0.018	0.026	0.015	0.023	–0.015	0.025	0.000	0.015	–0.002	0.017	0.001	0.015	–0.003	0.017	–0.001	0.014	0.001	0.016	–0.001	0.014	0.001	0.016
2	–0.016	0.023	0.021	0.027	0.032	0.039	–0.021	0.025	0.002	0.016	–0.002	0.016	0.001	0.016	–0.003	0.016	0.001	0.016	–0.001	0.012	0.001	0.015	0.000	0.013
3	–0.014	0.024	0.028	0.035	0.029	0.038	–0.024	0.029	0.006	0.019	0.000	0.016	0.006	0.020	–0.002	0.016	0.001	0.015	0.002	0.014	0.001	0.015	0.003	0.014
4	–0.007	0.015	0.012	0.026	0.023	0.029	–0.014	0.024	0.003	0.014	–0.003	0.020	0.003	0.015	–0.004	0.020	0.001	0.013	–0.001	0.018	0.001	0.013	0.000	0.018
5	–0.017	0.022	0.022	0.028	0.045	0.049	–0.027	0.033	0.007	0.017	–0.005	0.018	0.004	0.016	–0.007	0.018	0.001	0.012	–0.005	0.017	0.002	0.013	–0.003	0.016
6	–0.013	0.017	0.010	0.022	0.019	0.026	–0.015	0.020	0.000	0.013	–0.007	0.016	0.001	0.013	–0.007	0.016	0.000	0.011	–0.003	0.015	–0.001	0.011	–0.003	0.015
7	–0.019	0.027	0.026	0.033	0.025	0.036	–0.021	0.027	–0.001	0.021	0.002	0.018	–0.003	0.021	0.000	0.017	–0.005	0.018	0.000	0.014	–0.004	0.019	0.001	0.014
8	–0.026	0.034	0.025	0.031	0.026	0.031	–0.019	0.030	–0.001	0.016	0.000	0.020	–0.001	0.015	–0.002	0.020	–0.005	0.015	0.003	0.015	–0.006	0.014	0.004	0.015
9	–0.005	0.018	0.018	0.026	0.023	0.031	–0.010	0.021	0.004	0.017	0.003	0.018	0.005	0.017	0.002	0.018	0.002	0.016	0.004	0.017	0.002	0.016	0.005	0.018
10	–0.014	0.020	0.025	0.033	0.043	0.047	–0.028	0.034	0.010	0.018	–0.001	0.019	0.007	0.017	–0.003	0.019	0.006	0.015	0.001	0.016	0.007	0.015	0.002	0.016
11	–0.007	0.015	0.048	0.055	–0.007	0.019	0.533	0.533	–0.056	0.057	0.281	0.282	–0.056	0.057	0.277	0.278	–0.017	0.021	0.001	0.022	–0.018	0.022	0.005	0.023
12	–0.004	0.012	0.054	0.063	–0.006	0.022	0.529	0.529	–0.054	0.055	0.280	0.281	–0.055	0.056	0.276	0.277	–0.016	0.022	0.003	0.022	–0.016	0.022	0.007	0.024
13	–0.003	0.010	0.082	0.094	–0.004	0.025	0.694	0.694	–0.065	0.066	0.533	0.534	–0.065	0.065	0.528	0.528	–0.028	0.032	0.006	0.031	–0.026	0.030	0.016	0.036
14	–0.005	0.012	0.088	0.098	–0.003	0.020	0.695	0.695	–0.066	0.066	0.535	0.536	–0.066	0.066	0.530	0.531	–0.030	0.032	0.005	0.024	–0.027	0.029	0.018	0.031
15	0.530	0.530	–0.005	0.019	0.053	0.060	–0.007	0.014	0.278	0.279	–0.055	0.056	0.277	0.278	–0.055	0.056	–0.003	0.020	0.000	0.019	–0.001	0.020	–0.016	0.020
16	0.534	0.534	–0.009	0.018	0.072	0.076	–0.010	0.015	0.289	0.290	–0.057	0.058	0.288	0.289	–0.057	0.058	0.000	0.017	–0.002	0.018	0.004	0.018	–0.019	0.022
17	0.692	0.692	0.008	0.026	0.119	0.131	–0.001	0.008	0.533	0.534	–0.061	0.062	0.530	0.531	–0.061	0.062	0.012	0.035	0.014	0.030	0.018	0.040	–0.020	0.023
18	0.695	0.695	–0.003	0.024	0.120	0.131	–0.003	0.010	0.538	0.539	–0.064	0.065	0.536	0.536	–0.064	0.065	0.006	0.039	0.001	0.028	0.012	0.041	–0.026	0.029
19	0.254	0.255	0.009	0.022	0.004	0.020	0.259	0.260	–0.005	0.018	0.000	0.015	–0.007	0.019	–0.003	0.015	–0.008	0.020	–0.002	0.019	–0.006	0.020	0.001	0.019
20	0.256	0.257	0.009	0.025	0.015	0.022	0.256	0.257	0.004	0.017	0.002	0.019	0.002	0.017	–0.001	0.019	–0.001	0.016	–0.001	0.021	0.001	0.016	0.002	0.021
21	0.335	0.336	0.013	0.033	0.009	0.033	0.343	0.343	0.003	0.022	0.011	0.025	0.000	0.022	0.006	0.023	0.002	0.032	0.006	0.032	–0.004	0.026	0.003	0.026
22	0.345	0.346	0.010	0.030	0.012	0.034	0.333	0.333	0.010	0.026	0.001	0.023	0.006	0.025	–0.004	0.024	0.001	0.028	0.001	0.028	–0.002	0.025	–0.002	0.024
23	0.264	0.264	0.008	0.024	0.016	0.029	0.253	0.254	0.011	0.022	0.002	0.022	0.010	0.021	0.000	0.023	0.006	0.025	–0.001	0.021	0.007	0.025	0.002	0.022
24	0.257	0.258	0.006	0.022	0.012	0.021	0.253	0.253	0.004	0.017	–0.001	0.021	0.003	0.016	–0.004	0.021	–0.001	0.016	–0.003	0.021	0.000	0.016	0.000	0.021
25	0.346	0.346	0.011	0.032	0.008	0.038	0.331	0.332	0.009	0.030	0.003	0.025	0.005	0.029	–0.003	0.025	0.000	0.035	0.005	0.029	–0.003	0.031	0.001	0.024
26	0.338	0.339	0.005	0.031	0.013	0.030	0.339	0.340	0.009	0.024	0.008	0.033	0.006	0.023	0.002	0.031	0.002	0.025	–0.002	0.030	–0.001	0.023	–0.002	0.028
27	0.191	0.192	0.010	0.020	0.005	0.020	0.323	0.324	–0.024	0.028	0.038	0.043	–0.025	0.028	0.034	0.040	–0.001	0.019	–0.006	0.016	0.000	0.019	–0.002	0.015
28	0.191	0.192	0.019	0.031	0.010	0.024	0.324	0.324	–0.021	0.027	0.045	0.053	–0.023	0.028	0.041	0.050	0.002	0.023	0.004	0.025	0.002	0.023	0.007	0.026
29	0.171	0.172	0.032	0.046	0.011	0.030	0.512	0.512	–0.046	0.049	0.223	0.225	–0.047	0.050	0.217	0.220	0.002	0.023	0.007	0.031	–0.010	0.025	0.015	0.035
30	0.166	0.166	0.035	0.046	0.002	0.029	0.515	0.516	–0.050	0.052	0.226	0.228	–0.051	0.053	0.221	0.223	–0.005	0.024	0.011	0.032	–0.016	0.027	0.019	0.036

Note, RMSE = root mean square error; DINA = deterministic input, noisy "and" gate model; DINO = deterministic input, noisy "or" gate model; DINR = deterministic input, noisy ratio model; ACDM = additive cognitive diagnosis model; GDINA = generalized DINA model; DINMix = deterministic input, noisy mixed model; * = converted from the intercept and interaction parameters.

Table S4. Summary of the Item-Level –2LCPO of Six Models in Test Situation (f) in Simulation Study 2.

Analysis Model	Item														
	1	2	3	4	5	6	7	8	9	10	11	12	13	14	15
DINA	831.51	838.41	891.39	834.05	850.32	818.44	839.22	880.38	841.69	856.22	746.00	759.65	721.53	718.01	1138.96
DINO	830.28	857.40	894.42	856.65	875.88	834.07	850.51	896.67	859.90	876.99	1129.66	1133.25	964.80	962.95	764.62
DINR	782.30	804.13	874.24	800.70	822.72	775.61	805.32	867.66	808.16	829.94	942.63	951.03	880.41	875.65	951.07
ACDM	781.09	801.85	874.71	798.89	819.73	774.19	802.46	868.94	808.29	826.90	943.71	951.23	881.80	877.52	951.90
GDINA	725.23	736.61	803.57	734.39	741.20	719.46	726.38	797.28	745.34	756.61	712.11	724.98	697.68	692.17	725.64
DINMix	726.98	736.95	803.67	735.62	742.06	721.02	728.16	795.52	746.05	757.47	712.77	725.98	694.83	688.82	726.49
Analysis Model	Item														
	16	17	18	19	20	21	22	23	24	25	26	27	28	29	30
DINA	1129.30	975.34	961.75	1165.14	1163.37	1294.37	1296.91	1169.82	1162.73	1297.93	1291.78	1104.23	1112.52	1143.12	1137.01
DINO	775.02	754.55	746.42	1164.34	1173.74	1294.61	1294.91	1170.21	1168.27	1289.80	1298.39	1196.22	1202.77	1252.74	1246.85
DINR	948.63	893.32	880.20	1061.81	1073.24	1197.76	1197.71	1076.08	1070.97	1197.54	1202.71	1056.44	1069.52	1140.87	1134.20
ACDM	949.39	895.05	881.94	1061.96	1073.24	1200.19	1200.23	1058.81	1054.68	1186.78	1190.78	1053.83	1066.36	1141.58	1133.74
GDINA	728.51	721.54	710.07	1043.91	1053.63	1189.85	1190.30	1039.27	1034.12	1176.39	1178.90	1023.28	1037.89	1091.46	1084.07
DINMix	729.11	717.93	706.95	1042.95	1052.90	1185.86	1185.33	1057.75	1051.48	1185.97	1189.57	1026.19	1040.02	1095.02	1088.43

Table S5. The Estimated Item Parameters for the Fraction Subtraction Data (Posterior Mean).

Item	DINA		DINO		DINR		ACDM		GDINA		Wald-selected		DINMix				
	g_i	s_i	g_i	s_i	g_i	s_i	g_i*	s_i*	g_i*	s_i*	g_i	s_i	g_i	s_i	τ_{i1}	τ_{i2}	τ_{i3}
1	0.044	0.077	0.030	0.117	0.010	0.012	0.010	0.061	0.008	0.049	0.017*	0.099*	0.018	0.070	0.850	0.037	0.114
2	0.024	0.040	0.053	0.031	0.031	0.007	0.020	0.024	0.015	0.016	0.050	0.037	0.019	0.031	0.895	0.037	0.068
3	0.007	0.133	0.011	0.107	0.005	0.053	0.005	0.101	0.005	0.080	0.010	0.114	0.007	0.108	0.958	0.014	0.028
4	0.237	0.110	0.212	0.129	0.180	0.038	0.023	0.012	0.050	0.068	0.041*	0.113*	0.162	0.110	0.819	0.073	0.108
5	0.309	0.152	0.297	0.230	0.269	0.076	0.052	0.031	0.183	0.094	0.333	0.117	0.161	0.121	0.609	0.183	0.208
6	0.199	0.038	0.677	0.007	0.566	0.016	0.325	0.028	0.503	0.033	0.270	0.035	0.336	0.033	1	0	0
7	0.035	0.201	0.017	0.250	0.007	0.100	0.011	0.130	0.008	0.065	0.041	0.184	0.012	0.172	0.896	0.034	0.070
8	0.433	0.164	0.618	0.034	0.526	0.068	0.474	0.149	0.499	0.095	0.446	0.153	0.443	0.137	1	0	0
9	0.236	0.248	0.532	0.044	0.504	0.159	0.522	0.203	0.402	0.136	0.294	0.233	0.345	0.230	1	0	0
10	0.036	0.191	0.016	0.246	0.006	0.128	0.009	0.158	0.018	0.104	0.035	0.143	0.013	0.150	0.924	0.024	0.053
11	0.068	0.084	0.072	0.075	0.056	0.026	0.036	0.027	0.044	0.035	0.067	0.082	0.032	0.075	0.908	0.037	0.055
12	0.267	0.038	0.540	0.038	0.459	0.009	0.183	0.043	0.069	0.050	0.057*	0.043*	0.035	0.041	0.153	0.551	0.296
13	0.017	0.335	0.005	0.528	0.004	0.342	0.004	0.345	0.004	0.277	0.017	0.333	0.007	0.331	0.941	0.013	0.046
14	0.163	0.045	0.541	0.036	0.461	0.024	0.175	0.034	0.070	0.033	0.016*	0.038*	0.026	0.038	0.182	0.418	0.401
15	0.032	0.113	0.043	0.148	0.013	0.026	0.010	0.039	0.014	0.038	0.033	0.111	0.014	0.102	0.904	0.034	0.062
16	0.185	0.091	0.506	0.051	0.425	0.033	0.143	0.056	0.059	0.052	0.026*	0.081*	0.033	0.079	0.177	0.453	0.370
17	0.043	0.142	0.049	0.136	0.018	0.054	0.006	0.025	0.007	0.032	0.044	0.142	0.012	0.136	0.902	0.031	0.067
18	0.131	0.135	0.050	0.162	0.025	0.046	0.009	0.061	0.008	0.077	0.133	0.032	0.013	0.132	0.659	0.022	0.319
19	0.026	0.235	0.004	0.447	0.004	0.199	0.004	0.240	0.005	0.058	0.027	0.207	0.009	0.219	0.940	0.013	0.047
20	0.019	0.144	0.014	0.195	0.005	0.063	0.005	0.106	0.006	0.044	0.019	0.105	0.009	0.135	0.958	0.012	0.030

Note, g = guessing parameter; s = slip parameter; DINA = deterministic input, noisy "and" gate model; DINO = deterministic input, noisy "or" gate model; DINR = deterministic input, noisy ratio model; ACDM = additive cognitive diagnosis model; GDINA = generalized DINA model; DINMix = deterministic input, noisy mixed model; Wald-selected = selected mixing model via Wald test; * = converted from the intercept and interaction parameters.

Table S6. The Estimated Item Parameters for the Fraction Subtraction Data (Posterior Standard Deviation).

Item	DINA		DINO		DINR		ACDM		GDINA		Wald-selected		DINMix				
	g_i	s_i	g_i	s_i	g_i	s_i	g_i*	s_i*	g_i*	s_i*	g_i	s_i	g_i	s_i	τ_{i1}	τ_{i2}	τ_{i3}
1	0.016	0.018	0.016	0.019	0.010	0.011	0.010	0.032	0.008	0.053	0.017*	0.023*	0.016	0.019	0.065	0.030	0.073
2	0.015	0.012	0.017	0.012	0.017	0.006	0.012	0.010	0.013	0.009	0.016	0.012	0.015	0.011	0.053	0.027	0.054
3	0.007	0.021	0.010	0.019	0.005	0.018	0.005	0.019	0.005	0.020	0.007	0.019	0.007	0.020	0.027	0.013	0.025
4	0.026	0.021	0.025	0.022	0.027	0.021	0.025	0.013	0.048	0.058	0.034*	0.022*	0.055	0.021	0.095	0.064	0.087
5	0.029	0.024	0.032	0.025	0.031	0.027	0.048	0.028	0.073	0.095	0.030	0.023	0.073	0.024	0.128	0.108	0.154
6	0.054	0.010	0.026	0.006	0.032	0.008	0.052	0.010	0.093	0.011	0.055	0.010	0.051	0.009	0	0	0
7	0.014	0.028	0.011	0.028	0.006	0.031	0.010	0.035	0.008	0.042	0.015	0.027	0.011	0.029	0.050	0.025	0.056
8	0.049	0.020	0.027	0.016	0.033	0.016	0.043	0.021	0.037	0.034	0.045	0.021	0.043	0.020	0	0	0
9	0.058	0.023	0.027	0.034	0.031	0.030	0.029	0.027	0.048	0.035	0.057	0.023	0.048	0.023	0	0	0
10	0.012	0.028	0.009	0.029	0.006	0.032	0.008	0.031	0.013	0.098	0.012	0.029	0.011	0.029	0.035	0.018	0.037
11	0.015	0.019	0.016	0.018	0.016	0.015	0.019	0.019	0.016	0.025	0.015	0.018	0.020	0.018	0.043	0.027	0.042
12	0.048	0.012	0.031	0.016	0.035	0.008	0.047	0.021	0.073	0.047	0.057*	0.014*	0.033	0.014	0.091	0.137	0.185
13	0.008	0.031	0.005	0.028	0.004	0.035	0.004	0.033	0.004	0.083	0.009	0.031	0.007	0.032	0.031	0.012	0.031
14	0.039	0.012	0.029	0.013	0.033	0.011	0.048	0.011	0.042	0.014	0.015*	0.012*	0.023	0.012	0.116	0.146	0.224
15	0.016	0.023	0.015	0.026	0.010	0.019	0.010	0.026	0.012	0.026	0.015	0.023	0.012	0.024	0.055	0.029	0.055
16	0.037	0.017	0.028	0.016	0.033	0.013	0.042	0.015	0.031	0.019	0.025*	0.016*	0.025	0.016	0.117	0.153	0.229
17	0.013	0.022	0.014	0.022	0.010	0.022	0.007	0.023	0.008	0.032	0.013	0.023	0.011	0.023	0.040	0.022	0.046
18	0.021	0.023	0.017	0.024	0.016	0.026	0.010	0.041	0.008	0.077	0.021	0.028	0.012	0.023	0.067	0.021	0.073
19	0.009	0.032	0.004	0.029	0.004	0.041	0.004	0.039	0.006	0.075	0.010	0.032	0.008	0.033	0.027	0.012	0.029
20	0.009	0.026	0.008	0.026	0.005	0.029	0.005	0.034	0.006	0.057	0.009	0.025	0.008	0.026	0.023	0.010	0.023

Note, *g* = guessing parameter; *s* = slip parameter; DINA = deterministic input, noisy "and" gate model; DINO = deterministic input, noisy "or" gate model; DINR = deterministic input, noisy ratio model; ACDM = additive cognitive diagnosis model; GDINA = generalized DINA model; DINMix = deterministic input, noisy mixed model; Wald-selected = selected mixing model via Wald test; * = converted from the intercept and interaction parameters.

Figure S1. Root Mean Square Error of Attribute Profile Proportions in Simulation Study 2.

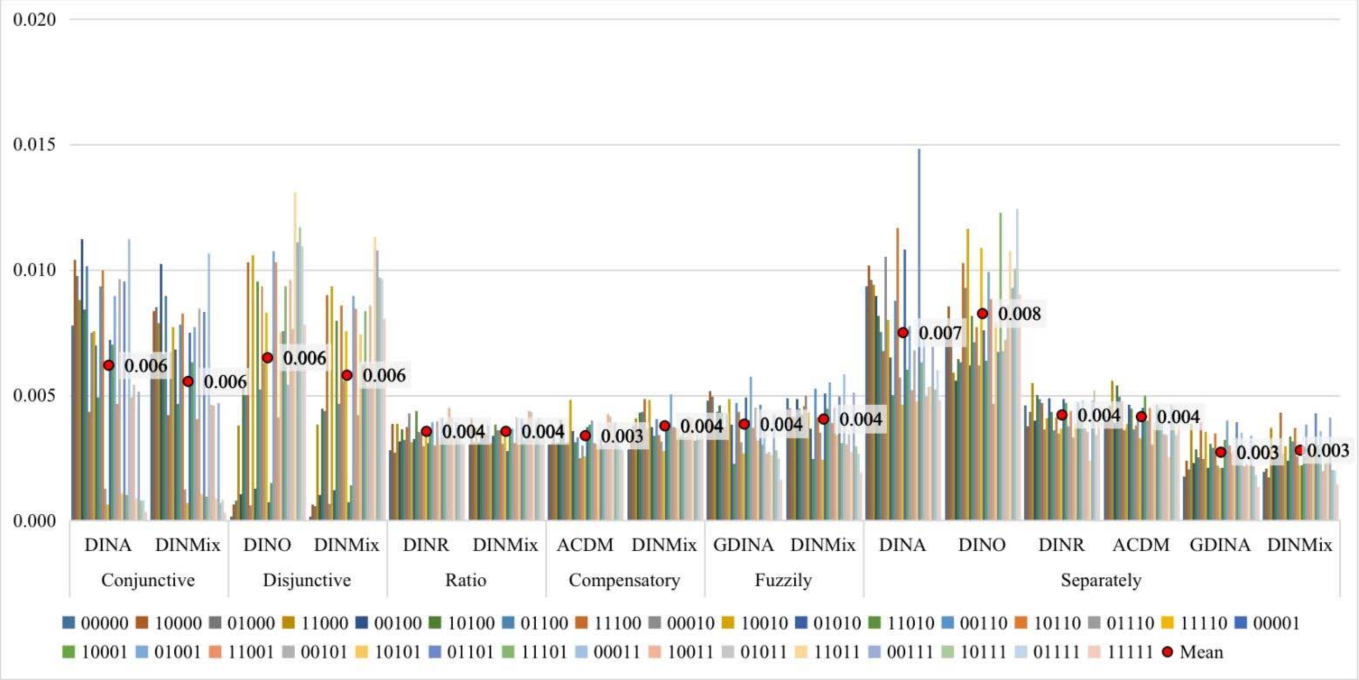

Note. DINA = deterministic input, noisy "and" gate model; DINO = deterministic input, noisy "or" gate model; DINR = deterministic input, noisy ratio model; ACDM = additive cognitive diagnosis model; GDINA = generalized DINA model; DINMix = deterministic input, noisy mixed model; Mean = mean root mean square error of 32 attribute profile proportions.
